# Supplementary figures and images for: Transcriptional repression by ApiAP2 factors is central to chronic toxoplasmosis
Source: PLoS Pathog. 2018 May 2;14(5):e1007035. doi: 10.1371/journal.ppat.1007035 (PMC5951591; doi:10.1371/journal.ppat.1007035)

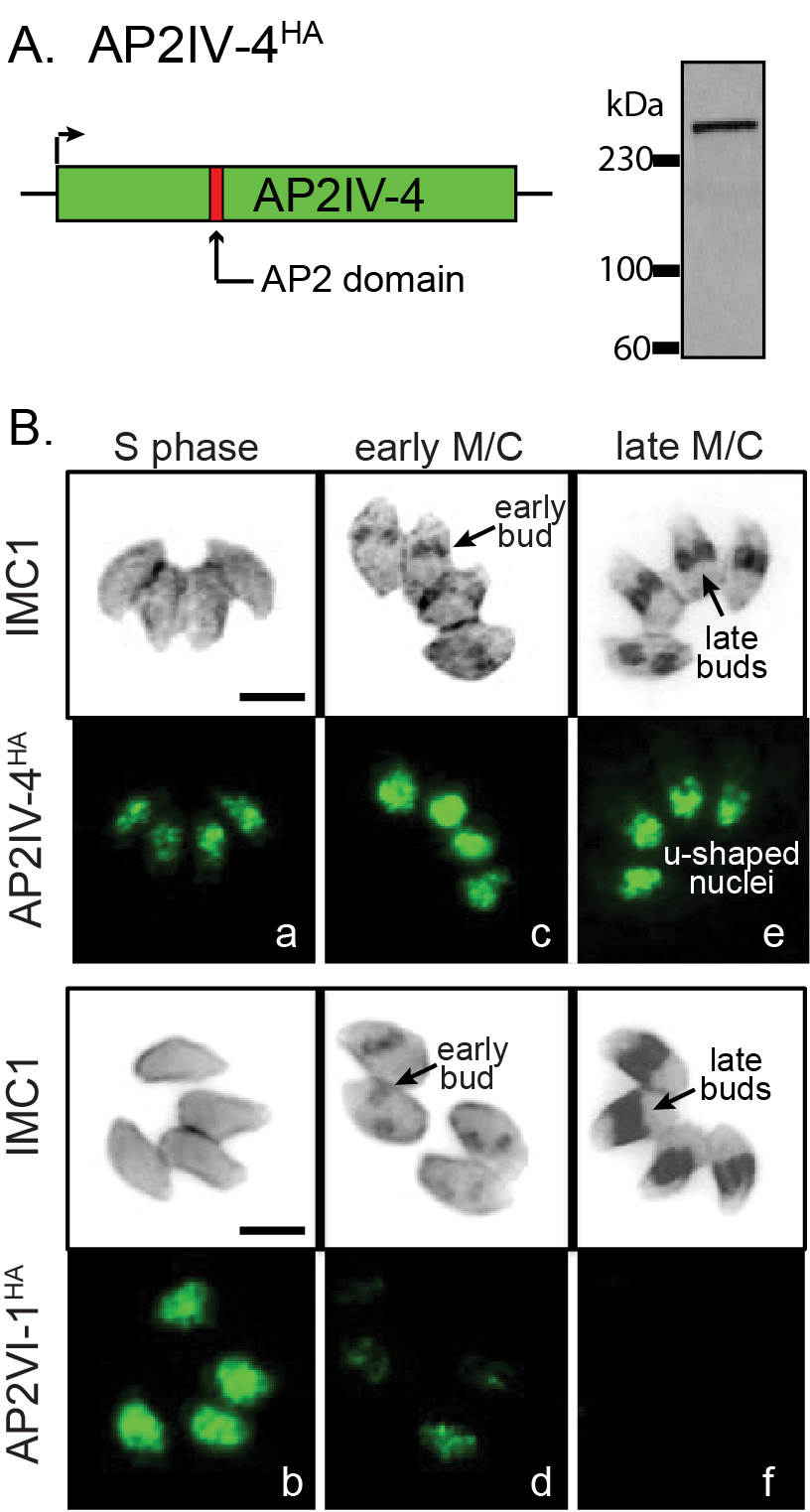

Supplement: S1 Fig — (A.) The AP2IV-4 single exon and AP2 domain gene is predicted to encode a ~250kDa protein (http://toxodb.org/toxo/app/record/gene/TGME49_318470), which was confirmed by Western analysis (α-HA) of AP2IV-4HA parasites (plus 3xHA, actual protein size is ~254kDa). Molecular mass standards indicated on the left. (B.) IFA analysis of AP2IV-4HA and AP2VI-1HA transgenic parasites grown in HFF monolayers utilized co-stains α-HA (green, AP2IV-4HA or AP2VI-1HA expression) and α-IMC1 (black and white panels). To improve visualization of internal bud structures, IMC1 images were decolorized and then inverted. AP2IV-4HA and AP2VI-1HA are exclusively localized to the nucleus and the relative cell cycle expression profile predicted by the cyclical mRNA patterns (Fig 1A, green and red curves) was confirmed for each factor. Black scale bar = 5 μm. (TIF) [file ppat.1007035.s004.tif]

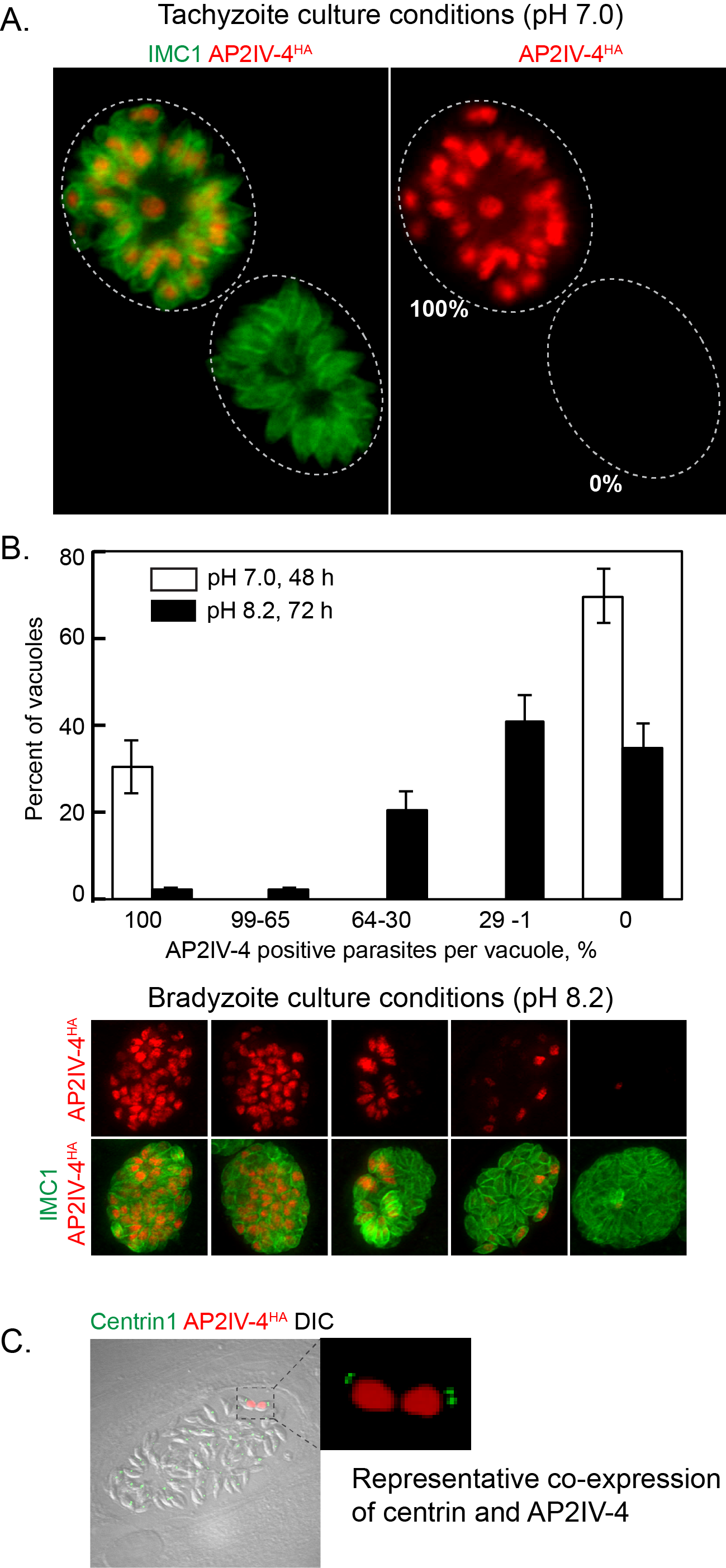

Supplement: S2 Fig — (A.) Representative microscope field showing two vacuoles of PruQ-AP2IV-4HA parasites; one vacuole is synchronously expressing AP2IV-4HA, while the other is negative for this factor due to the cell cycle regulation of AP2IV-4 expression. IFA analysis utilized co-stains α-HA (red, AP2IV-4HA) and α-IMC1 (green). (B.) Quantification of AP2IV-4HA expression in tachyzoites and alkaline-shifted bradyzoites at the times indicated. 100 randomly selected vacuoles stained with α-HA (red, AP2IV-4HA) and DBA were counted in triplicate. Tissue cysts (DBA+) that were also positive for AP2IV-4HA were sorted into five bins based on the fraction of AP2IV-4HA positive parasites. IFA images showing representative patterns of AP2IV-4HA expression in the tissue cysts from each of the five bins (cell cycle marker IMC1 and AP2IV-4HA co-stains) is shown below the graph. (C.) Representative image of a differentiating vacuole (72 h post-alkaline shift) co-stained with α-centrin (centrosome) and α-HA (AP2IV-4HA). Note the distinct morphology of the two AP2IV-4HA parasites that possessed a duplicated centrosome adjacent to the nucleus, which are likely mitotic. In contrast to the uniform centrin staining in the AP2IV-4HA-positive parasites, centrin staining in the AP2IV-4HA-negative parasites varied in intensity and composition with many parasites containing a faint, single dot of centrin. (TIF) [file ppat.1007035.s005.tif]

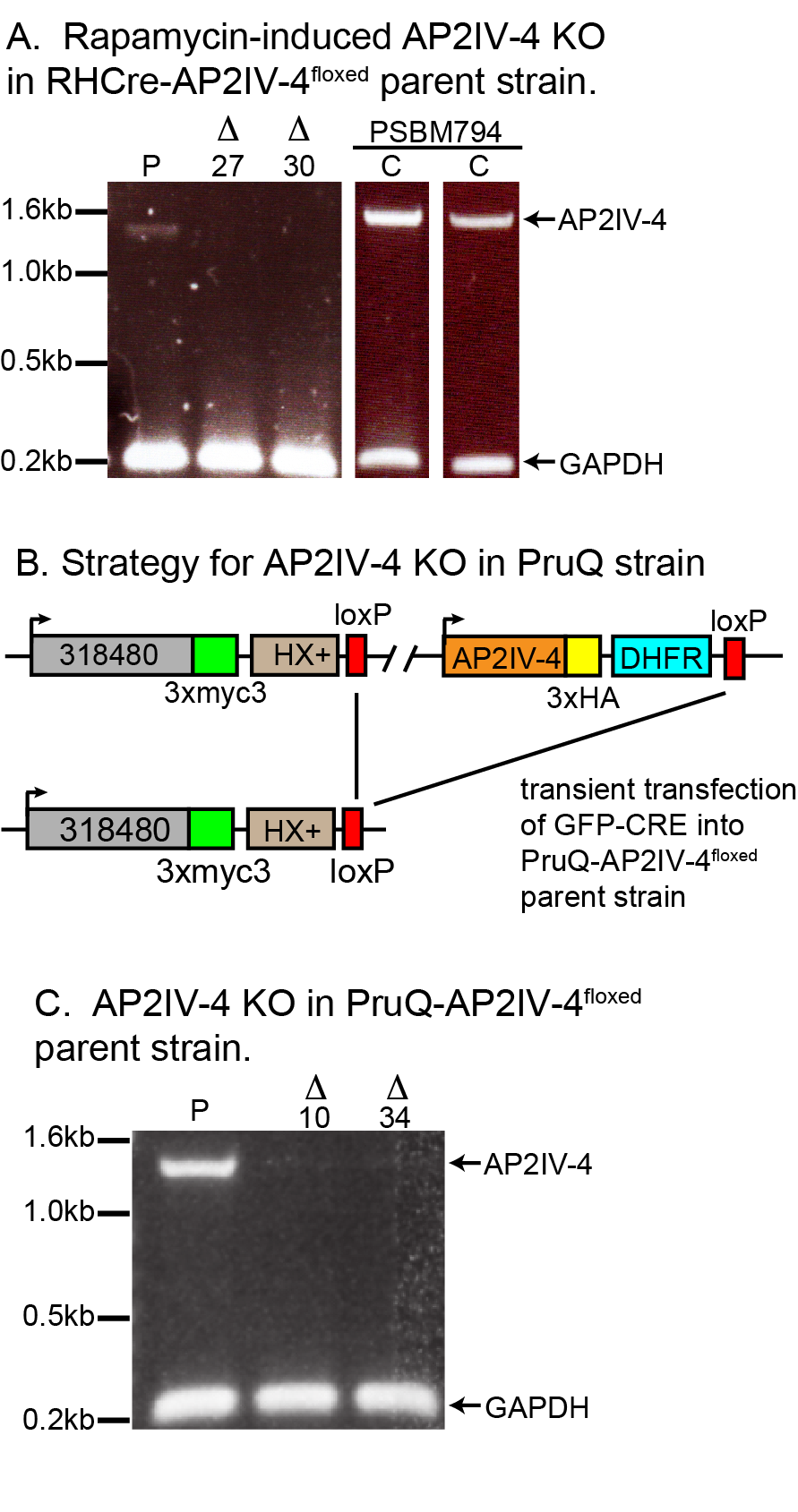

Supplement: S3 Fig — (A.) Verification of AP2IV-4 knockout by RT-PCR in the RHCre-Δap2IV-4 transgenic clones. AP2IV-4 mRNA is detected in lane P (parent strain, RHCre-AP2IV-4floxed), but absent from both AP2IV-4 knockout clones evaluated (RHCre-Δap2IV-4 clones 27, and 30). For a diagram of the RHCre knockout strategy see Fig 3A. Complementation for the loss of AP2IV-4 in RHCre-Δap2IV-4 parasites was accomplished using cosmid PSBM794, which reintroduces a full copy of the AP2IV-4 gene and restores AP2IV-4 mRNA expression (C lanes). (B.) Diagram of the AP2IV-4 knockout strategy in Type II PruQ-parent (AP2IV-4floxed), which is similar to the knockout of AP2IV-4 in the RHCre strain (see Fig 3A). Here transient transfection of pMIN-CRE-eGFP plasmid into the PruQ-AP2IV-4floxed strain was required to introduce active Cre recombinase. (C.) PruQ-parent (lane P) expresses the expected AP2IV-4 transcript, whereas PruQ-Δap2IV-4 clones 10 and 34 (lanes Δ10 and Δ34) lack the AP2IV-4 mRNA. AP2IV-4 cDNA primers amplify a 1367bp fragment of the single exon gene (7221bp full length). For loading and template quality control, GAPDH primers were designed around a 432bp intron, allowing detection of both mRNA (202bp, intron spliced out) and contaminating genomic DNA (634 bp). The absence of a detectable 634bp amplicon in all samples indicates the RNA templates were free of genomic DNA contamination. See S3 Dataset for all primer designs. (TIF) [file ppat.1007035.s006.tif]

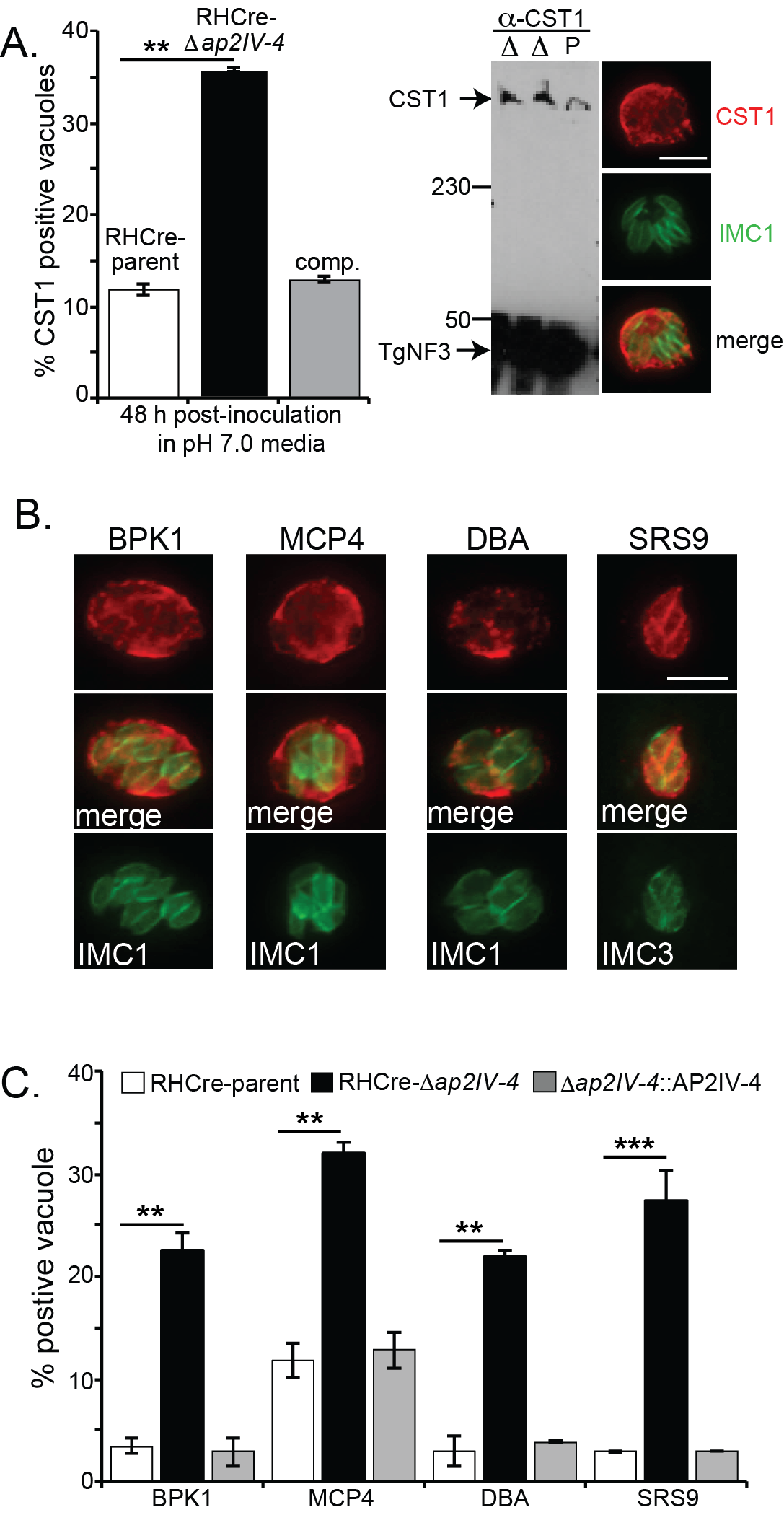

Supplement: S4 Fig — (A.) RHCre-parent (AP2IV-4floxed, open bar), -Δap2IV-4 knockout (black bar), and -Δap2IV-4::AP2IV-4 complemented (grey bar) parasites were grown in HFF cells for 24 h under standard tachyzoite culture conditions (pH 7.0) and then IFA analysis performed using α-IMC1 (green stain, tachyzoite replication marker) and antibodies to cyst wall protein, CST1 (red stain). Scale bar = 5 μm. CST1 positive vacuoles in each strain were quantified in triplicate by counting 100 vacuoles in randomly selected microscopic fields (**, p<0.01). Increased expression of CST1 protein in two independent RHCre-Δap2IV-4 clones (Δ clones, see also S3A Fig) compared to the RHCre-parental (P = AP2IV-4floxed) strain revealed by Western analysis. Nucleolar TgNF3 (43kDa) protein was included as a loading control. Protein mass markers 230kDa and 50 kDa on left. Immunofluorescence images included on right are representative of CST1 positive vacuoles showing cyst wall localization in RHCre-Δap2IV-4 parasites. (B.) Representative IFA images of RHCre-Δap2IV-4 tachyzoites expressing bradyzoite-specific proteins BPK1 and MCP4 with normal localization at the periphery of the vacuole consistent with DBA+ cyst wall structures, likewise expression of bradyzoite-specific SRS9 in the RHCre-Δap2IV-4 tachyzoites was properly localized to the parasite surface. Note the uniform intravacuolar expression of SRS9 in RHCre-Δap2IV-4. Scale bar = 5 μm. (C.) Numbers of BPK1, MPC4, DBA, and SRS9 positive vacuoles in RHCre-parent, RHCre-Δap2IV-4 and RHCre-Δap2IV-4:AP2IV-4 tachyzoites. Statistical significance indicated (**, p<0.01; ***, p<0.001). Positive staining vacuoles for each strain were quantified in triplicate by counting 100 vacuoles in randomly selected microscopic fields. (TIF) [file ppat.1007035.s007.tif]

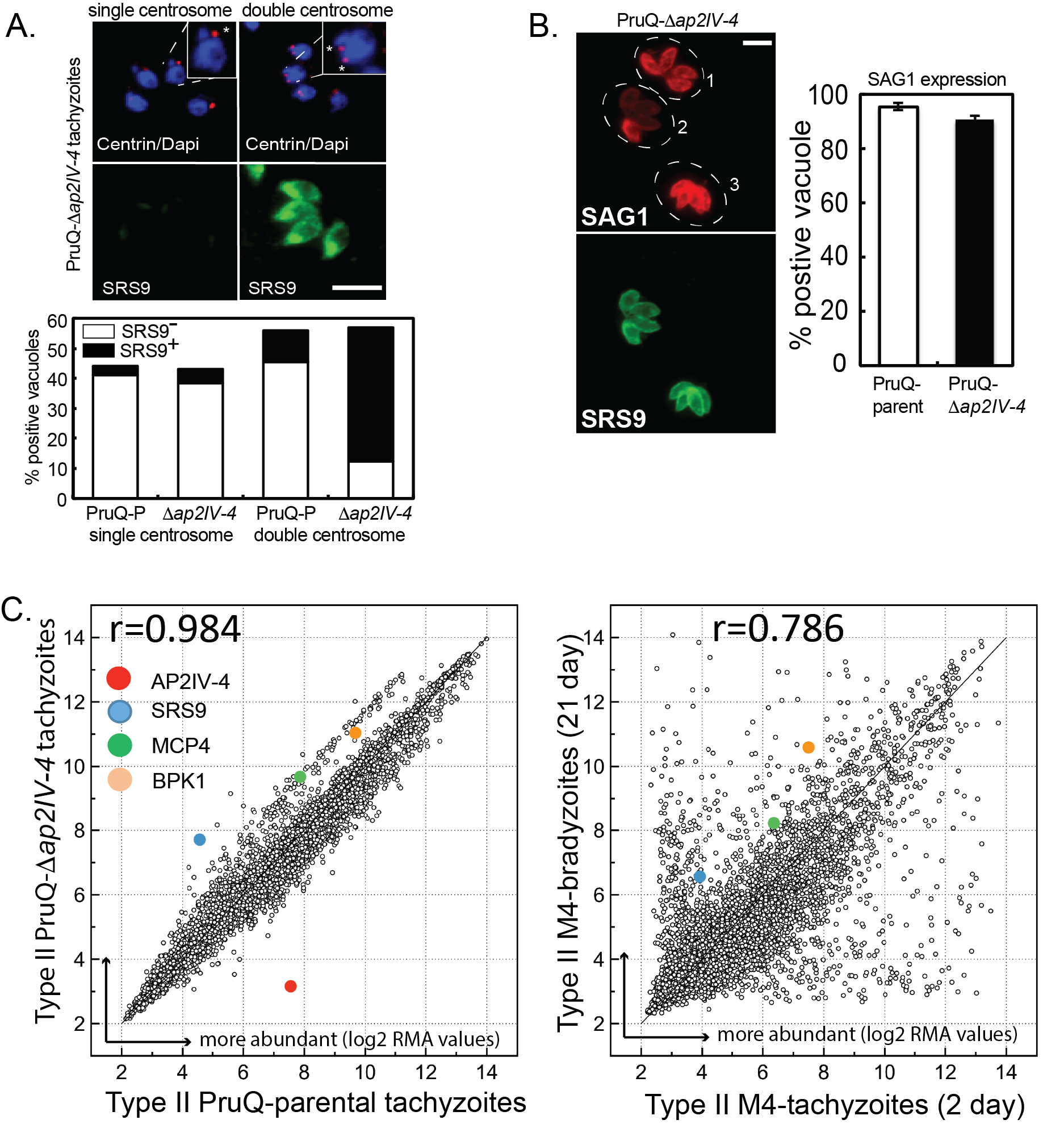

Supplement: S5 Fig — (A.) The cell cycle distribution of SRS9+ parasites in PruQ-Δap2IV-4 versus the PruQ-parent strains was determined by IFA analysis with αSRS9 and αCentrin (centrosome marker) antibodies. Representative PruQ-Δap2IV-4 co-stained images demonstrate the association of SRS9 expression with parasites containing duplicated centrosomes (S/M/C phases) that was quantified and graphed for PruQ-Δap2IV-4 and the PruQ-parent strains. All parasite counts were determined in triplicate from 100 vacuoles selected at random. The association of SRS9+ expression in S phase and mitotic PruQ-Δap2IV-4 parasites (duplicated centrosome) versus G1 phase (single centrosome) parasites was statistically significant (p<0.001). Scale bar = 5 μm. (B.) The tachyzoite nature of SRS9+ PruQ-Δap2IV-4 parasites was confirmed by IFA analysis with αSRS9 and αSAG1 antibodies. The expression of SRS9 in PruQ-Δap2IV-4 parasites was nearly ~50% (as shown in A.), and thus, about half the population was SAG1+/SRS9- (#1 vacuole of four parasites circled). Nearly all PruQ-Δap2IV-4 parasites expressing SRS9+ were also positive for SAG1+ surface expression (representative vacuole #3 circled). The fraction of SAG1+ positive vacuoles in PruQ-parent and PruQ-Δap2IV-4 (SRS9- and SRS9+) infected HFF cell cultures were quantified (see graph). In rare examples, PruQ-Δap2IV-4 parasites that were SRS9+ also showed diminished SAG1 expression (#2 vacuole circled). Scale bar = 5 μm (C.) Whole-cell mRNA analysis of Type II PruQ-Δap2IV-4 parasites. The analysis of total mRNA expression of PruQ-Δap2IV-4 versus PruQ-parent parasites grown under tachyzoite conditions shows nearly complete identity (r = 0.984) with the exception of the few mRNAs altered by the loss of AP2IV-4 (Fig 3A and S1 Dataset). Note mRNAs misexpressed by the loss of AP2IV-4 have higher levels in the PruQ-Δap2IV-4 parasites, while few tachyzoite mRNAs are downregulated. A reference plot for native Type II strain (M4 isolate) differences (r = 0.786) in development [file ppat.1007035.s008.tif]

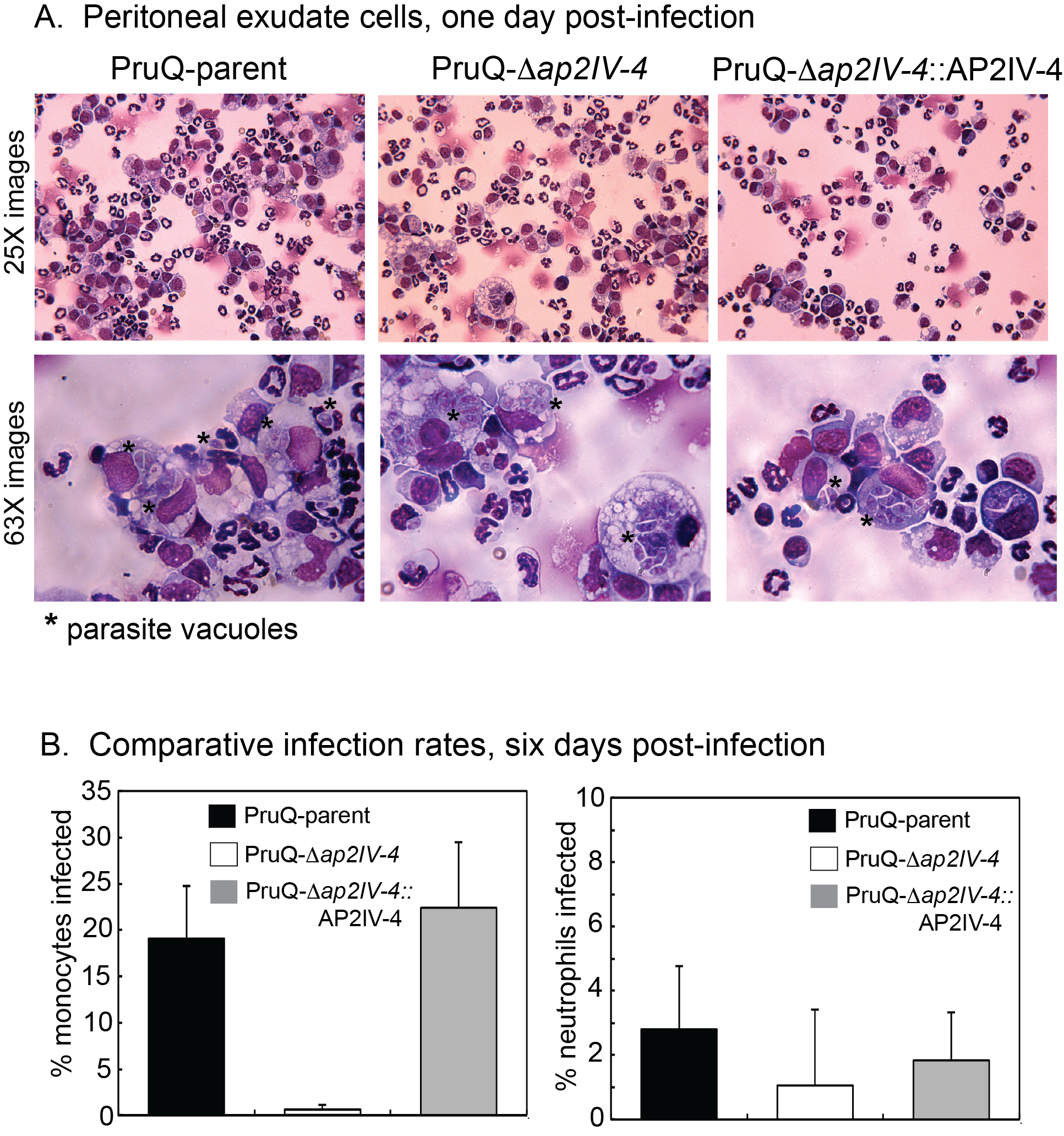

Supplement: S6 Fig — (A.) Peritoneal exudate cells were collected at one day post-infection with 107 tachyzoites intraperitoneally. Cells from the peritoneal wash were spun onto cytoslides and stained hematologically. Representative images at both 25X and 63X are provided. Representative parasite vacuoles are marked by a (*). (B.) Cytospins of peritoneal washes from animals six days post-infection were harvested and stained as above. Percentages of infected monocytes and polymorphonuclear neutrophils were determined by microscopy. (TIF) [file ppat.1007035.s009.tif]
